# Ascariasis Resulting from Swine-to-Human Transmission in Okinawa, Japan

**DOI:** 10.4269/ajtmh.22-0151

**Published:** 2022-05-16

**Authors:** Takeshi Kinjo, Hiromu Toma, Jiro Fujita

**Affiliations:** ^1^ Department of Infectious, Respiratory, and Digestive Medicine, Graduate School of Medicine, University of the Ryukyus, Okinawa, Japan;; ^2^ Department of Immunology and Parasitology, Graduate School of Medicine, University of the Ryukyus, Okinawa, Japan

**Figure 1.**
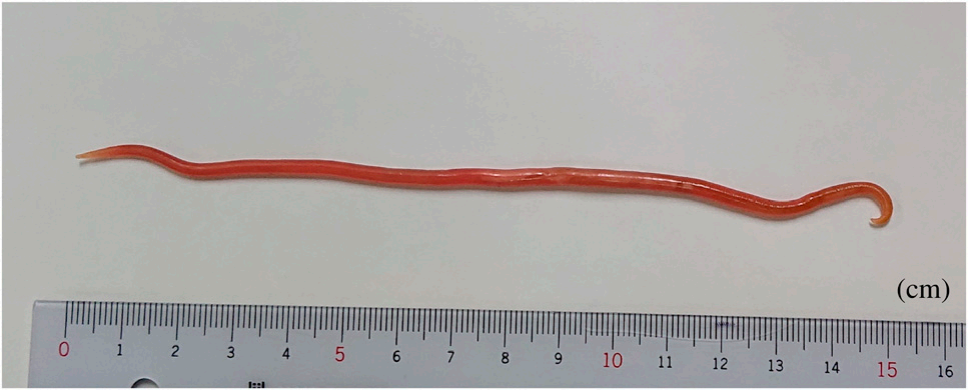
Adult male *Ascaris* from the patient. The size and shape (incurvated rear end and absence of genital hook) indicate that this is an adult male *Ascaris*. This figure appears in color at www.ajtmh.org.

**Figure 2.**
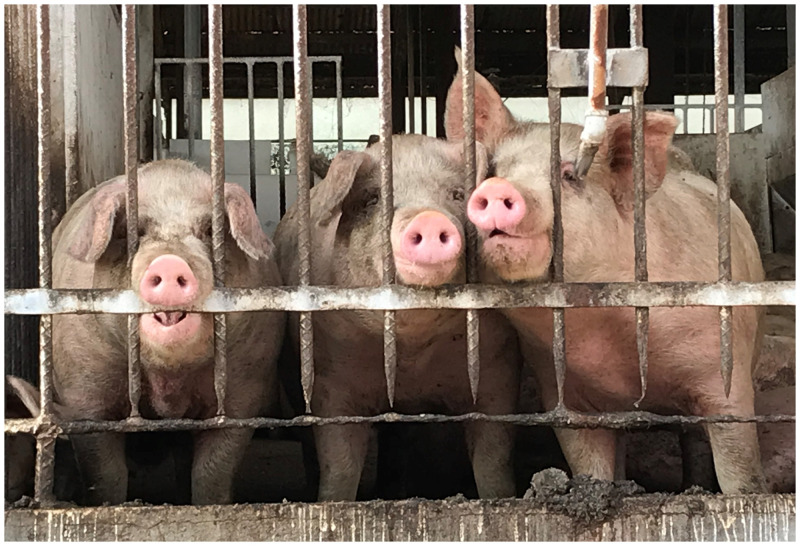
Pigs raised in the pigsty beside the patient’s farm. Swine-to-human transmission can occur via pig feces contaminated with *Ascaris* eggs. This figure appears in color at www.ajtmh.org.

A 67-year-old sugarcane farmer visited our hospital with a nematode infection (Figure 1) after having an itchy sensation in his anus the previous night and finding the nematode coming out of his anus that morning. He reported no history of overseas travel or symptoms, except weight loss of 5 kg over several months. No abnormalities were found on physical examination, in laboratory findings (including blood eosinophil counts and IgE levels), and in chest/abdominal computed tomography. No eggs were found in the stool. The nematode was identified morphologically as an adult male *Ascaris*. Two days after treatment with a single dose of 500 mg pyrantel pamoate, he found another 10-cm-long *Ascaris* worm excreted in his feces. His farm, located next to a pigsty (Figure 2), had been fertilized with manure from pigs 4 years, 2 years, and 5 months prior to the hospital visit. In addition, he often ate lunch without proper handwashing and may have acquired the infection through oral contact with his fingers contaminated with *Ascaris* eggs. Genome sequencing results for two polymorphic sites in the ribosomal RNA internal transcribed spacer 1 region—C and A in positions 133 and 246, respectively—were identified as those of *Ascaris suum*, suggesting pig origins.^1^^,^^2^

Ascariasis in humans is usually caused by *Ascaris lumbricoides.* However, *A. suum*, a parasite of pigs, has caused human infections in the United States and a few European countries.^3^^–^^5^ Although human-to-human transmission of *A. lumbricoides* is less likely in regions where sewerage systems are developed, swine-to-human zoonoses by *A. suum* can occur even in developed countries without adequate management of swine manure or control of swine parasites. This case highlights the importance of acknowledging *Ascaris* as an important zoonotic pathogen as well as the “One Health” approach to control infectious diseases.
